# Psychopathology and impairment of quality of life in offspring of psychiatric inpatients in southern Brazil: a preliminary study

**DOI:** 10.1186/s13034-018-0251-2

**Published:** 2018-10-20

**Authors:** Ana Luiza Ache, Paula Fernandes Moretti, Gibsi Possapp Rocha, Rogéria Recondo, Marco Antônio Pacheco, Lucas Spanemberg

**Affiliations:** 1 Núcleo de Formação em Neurosciências da Escola de Medicina da Pontifícia, Universidade Católica do Rio Grande do Sul, Av. Ipiranga 6690, Porto Alegre, CEP 90619-900 Brazil; 2 Hospital São Lucas da Pontifícia Universidade Católica do Rio Grande do Sul, Av. Ipiranga 6690, 6º andar sul, Porto Alegre, CEP 90619-900 Brazil

**Keywords:** Child development, Quality of life, Children psychiatric inpatients, Parent–child relations, Psychopathology

## Abstract

**Objective:**

To evaluate the quality of life and risk of psychopathology in the infant and adolescent offspring of psychiatric inpatients from a general hospital unit.

**Methods:**

Offspring (4–17 years old) of psychiatric inpatients were interviewed face-to-face and assessed with the Strengths and Difficulties Questionnaire (SDQ). Interviews with caregivers and the hospitalized parents were also performed. The quality of life of the offspring, psychopathology of their hospitalized parents, and their current caregivers were investigated in order to evaluate any associations between these aspects and psychopathology in the offspring.

**Results:**

Thirty-four children of 25 patients were evaluated, 38.2% of which presented high risk for some type of psychopathology including hyperactivity or attention deficit disorder (38.2%), behavioral disorders (20.6%), and emotional disorders (17.6%). While only the minority of these children (17.6%) were already receiving mental health treatment, another 41.2% of them exhibited some degree of symptoms and were only referred for specialized assessment. Additionally, 61.8% of the children were reported to be suffering from some impairment in their quality of life.

**Conclusion:**

This preliminary study found a high rate of psychopathology in children of psychiatric inpatients. These results corroborate previous evidence that children and adolescents with parents with severe psychopathology are at high risk for developing mental disorders. Public policies and standard protocols of action directed to this population are urgently needed, especially for offspring of parents that are hospitalized in psychiatric in-patient units of general hospitals.

## Background

Mental disorders represent a group of pathologies that have the greatest impact on global health burden. Recent findings have demonstrated that the global burden of mental illness accounts for 32.4% of years lived with disability (YLDs) and 13.0% of disability-adjusted life-years (DALYs) [1]. Most mental disorders begin in childhood. Moreover, it is reported that around 50% of mental disorders start before the age of 14 and 75% start before the age of 24 [2]. Thus, prevention and early identification of vulnerable children with psychopathology has been reported as the most effective strategy for reducing the implications and burdens of mental illness [3].

The prevalence of mental disorders in childhood has been increasing, ranging from around 13.4%, in community surveys around the world [4], up to 49% in clinical populations [5]. The US prevalence of youths with serious emotional disturbance with global impairment is about 6.36% [6]. In Brazil, studies have reported a prevalence of 30% of common mental disorders in adolescents [7] with 50% of adult mental disorders beginning before the age of 18 years [8]. In younger children, a prevalence of 13% of psychiatric disorders was found among 6-year-old children in a birth cohort in southern Brazil [9].

The children of patients with psychiatric disorders are a particularly vulnerable population for the development of psychopathology. Several studies have reported that the offspring of parents with mental problems are up to 13 times more likely to develop the same psychopathology [10–12] and are up to five times more likely to use professional mental health services [13, 14]. In addition, they have a higher risk of criminal convictions [15], self-harm [16], and violence and suicide [17, 18]. Data from the World Health Organization (WHO) World Mental Health Survey estimate that the population-attributable risk proportion for parent disorders is 12.4% across all offspring disorders [19]. Furthermore, it is estimated that about 15.6% of children in Canada are exposed to parents or guardians with psychopathology [20]. In Australia, 14.4% to 23.3% of children have a parent with some non-substance related mental disorder [21, 22]. In the US, the US National Survey of Drug Use and Health (2008–2014) reported that 2.7 million parents (3.8%) and 12.8 million parents (18.2%) had presented a serious mental illness or any mental illness in the past year, respectively [23]. Moreover, data appointed that up to 58% of children with serious emotional disorders have a history of family mental illness and 40% have a history of parent psychiatric hospitalization [24].

Despite the prevalence and the incredibly increased risk for negative outcomes in children of people with mental disorders, this population is often under-detected as well as poorly monitored and treated. A UK community study found that only 37% of children with any psychopathology and children of parents with depression had some recent contact (previous 3 months) with some assistance, of which only 15.2% had contact with a mental health service [25]. Estimates in Brazil are not clear, but a recent survey found that only a small proportion of children or adolescents with any psychiatric disorder (19.8%) were seen by a mental health specialist in the previous 12 months [26]. In addition, children of psychiatric patients, particularly those with severe mental disorders and a history of hospitalizations, present a higher risk of mortality, especially in early childhood and late adolescence [27]. Mothers with mental disorders lose custody or contact with their children more frequently [28]. Moreover, there is no routinization or systematization of mental health evaluations for the children of hospitalized patients. The training of professionals, adequacy of physical area and environments, and psychoeducation aimed at the promotion of children’s mental health and prevention of mental disorders are rare and frequently absent in the routines of hospitals, training programs, [29–31], and government policies [24].

Although more than 90% of the world’s children and adolescents live in low- and middle-income countries (LMICs), studies on high risk children are rare in these countries. Despite some population surveys, there are few, if any, studies in Brazil that have evaluated high-risk children of hospitalized psychiatric patients. The aim of this study was to investigate the prevalence of mental disorders and the impact on the quality of life in children of inpatients from a psychiatric unit of a general hospital in southern Brazil.

## Methods

### Sample and design

This was a cross-sectional observational study in which children were sampled over a period of 20 months (from April 2016 to November 2017). The study was carried out at the Psychiatric Inpatient Unit of the São Lucas Hospital, Pontifícia Universidade Católica do Rio Grande do Sul (HSL/PUCRS), a nonprofit university general hospital with 21 psychiatric beds. During the period, were admitted 399 inpatients (420 admissions). The average length of stay is about 30 days, and the average occupancy rate was 85% in the period. Many patients with extreme age (83 elderly and 33 adolescents), did not have children in the study’s age group, as well as others 204 adults (an indefinite number of these with dubious or unavailable data). A total of 79 patients had children in the study’s age group, although we only had information about the children in 66 cases (97 children). The cases that remained less than 7 days (7 patients, with 10 children) were not interviewed. The final eligible sample was 59 parents of 87 children. We were unable to contact or could not include 53 children (34 parents) for many reasons (such as lack of financial conditions to come to the hospital, the caregiver did not agree with the participation of the children, adopted children, etc.).

## Instruments

### Clinical and Sociodemographic Questionnaire (CSQ)

This questionnaire was part of the research protocol and contained data about clinical records and interviews with patients, their children, and families. It included questions about parents, caregivers, and their children, such as age, sex, marital status, occupational status, family income, number of people in the house, who the caregiver is during parent hospitalization, and characteristics of the hospitalized parent. In addition, data was collected from routine evaluations of the inpatients selected for the research, such as the psychiatric diagnosis as codified by International Classification of Diseases (ICD-10) after clinical interview.

### Strengths and Difficulties Questionnaire (SDQ)

This was a short questionnaire to screen for changes in the behavior of children aged 4–17 with both parent and educator versions. SDQ has become the most widely used research tool for the detection of mental health problems [32] and is currently available in more than 40 languages, including Portuguese. It had 25 items, from which 10 were related to capacities, 14 were about difficulties, and one was neutral item. These items were divided into five subscales for which each one was represented by five statements, namely emotional symptoms, behavioral problems, hyperactivity, relationship problems with colleagues, and pro-social behavior. The instrument was presented in three versions, and was intended to be answered by the children themselves (above 11 years), their parents or guardians, and teachers. There were several answer options: false (zero point for this type of response), plus or less true (one point), and true (two points). Only one option could be selected per item. For each of the five subscales, the score could range from 0 to 10. We proposed that the SDQ would be a promising alternative within the Brazilian scenario where standardized instruments for the evaluation of children’s mental health were scarce [32]. For this article, the SDQ individual scores were calculated in the official online website of the questionnaire [33]. This procedure was used to calculate all the dimensions of the instrument, as well as to internalize and externalize symptoms scores and the diagnostic predictors for psychopathology.

### Patient Health Questionnaire for Depression and Anxiety (PHQ-4)

The PHQ-4 is an ultra-brief screener for depression and anxiety. Health care staff can administer it or it can be self-administered [34]. A recent study found that higher PHQ-4 scores were strongly associated with functional impairment, disability days, and health care [35]. Total score was determined by adding together the scores for each of the four items. Scores are rated as normal (0–2), mild (3–5), moderate (6–8), and severe (9–12). The PHQ-4 is only a screening tool and does not diagnose depression.

### Mood Disorder Questionnaire (MDQ)

The MDQ is a short, single-page, paper and pencil self-report screening instrument for bipolar spectrum disorders for adults. It was divided into three sessions. The first session included 13 Yes/No questions derived from the DSM-IV criteria and clinical experience. The second asked whether several symptoms have been experienced in the same period of time. The third part examined psychosocial impairment, classified as absent, minor, moderate or serious. In the original validation study [36], MDQ positive screening for BDs required that seven or more positive symptoms be reported, with clustering within the same time period and causing moderate to severe problems. The Brazilian version of MDQ was previously demonstrated to be a valid instrument for the screening of bipolar disorders [37].

### Quality of Life Evaluation Scale (AUQEI)

This is a quality of life scale developed by Manificatet al [38] and was translated and validated for Brazilian language and culture in children aged from four to 12 years old. This instrument aimed to assess the subjective feeling of well-being by assuming that the developing individual is, and always has been, able to express himself or herself with respect to his or her own subjectivity. The questionnaire was based on the point of view of the child’s satisfaction. It had 26 questions covering the domains autonomy, leisure, functions, and family. To facilitate the application and comprehension, the questionnaire used images of four faces that expressed different emotional states. It allowed each child to understand the situations and present their own experience. The scale thus allowed us to obtain a profile of their satisfaction in different situations. It was validated in Brazil with children between 4 and 12 years and exhibited a cutoff point of 48 points for characterizing impairment in quality of life [38]. In order to calculate Z and T scores, we used Brazilian study averages as normative values (50.5 (± 3.5) and 53.5 (± 8.0) for boys and girls, respectively).

### The World Health Organization Quality of Life—short version (WHOQOL-BREF)

This instrument evaluates a patient’s quality of life and consists of 26 questions, with answers that use a Likert scale (from 1 to 5, the higher the score the better the quality of life). Apart from the first two questions, the instrument has 24 facets that comprise four domains: physical, psychological, social relations, and environment. Psychometric properties were analyzed using cross-sectional data obtained from a survey of adults carried out in 23 countries [39]. The WHOQOL-BREF Portuguese version was validated with high internal consistency (Cronbach’s alpha from .71 to .84 for the four domains), high test re-test reliability, satisfactory features of discriminant, as well as criterion and concurrent validity [40]. In order to calculate Z and T scores, we used the averages of the validation study as normative values by age groups in each domain [39].

### The Clinical Global Impression Scale-Severity (CGI-S)

This is a widely-used assessment tool in psychiatry, is easy to apply and interpret, and is available in the public domain [41]. The CGI-s assesses the degree of patient severity in relation to its psychopathology. Scores range from 1 (normal, not ill) to 7 (among the most severely ill patients). It was routinely used for inpatient assessment and its scores were recorded in the medical records.

## Procedures

We collected information from each study group with the following procedures:

- *Inpatients with children* The data about admission and medical and psychiatric history were collected from clinical records. The severity of psychopathology of the inpatients was measured by the clinical staff in the routine evaluation by the CGI-S scale. The patient’s psychiatric diagnosis was made by the patient’s physician, using International Classification of Diseases (ICD-10) after a clinical interview.
- *Main caregivers* All caregivers answered the CSQ with general information, as well as questions about the clinical aspects of the parent (e.g., number of previous hospitalizations, previous psychiatric treatment and initial psychiatric diagnosis) and questions about the children (e.g., years of study, difficulties before and during the parental hospitalization). Additionally, the caregivers answered the SDQ to screen for changes in the behavior of children; and the PHQ-4 and the MDQ scales, to identify symptoms of anxiety, depression, and bipolar disorder.
- *Offspring of psychiatric inpatients* All children were interviewed clinically for the first researcher (A.L.A.) in order to identify psychopathology in risk factors which could indicate the need for emergency intervention. The quality of life questionnaires were answered according to the child age; children 4–11 years old only answered questions from the AUQUEI and children older than 12 years old answered the WHOQOL-BREF. The SDQ (adolescent version) was answered by the children aged 11–17 years.

## Ethical considerations

The research protocol was submitted and approved by the Research Ethics Committee of the São Lucas Hospital of PUCRS (protocol number: 1.438.973) prior to the start of data collection. The participants received a consent term for the caregiver, the term of the consent for minors which was signed by the legal responsible for the children, and the term of assent, which was signed by the minors. All data was kept confidential, except when they constituted risk situations. Cases of children identified with psychopathology were referred for treatment. One case was identified as an emergency situation (suicidal ideation) and referred for assistance in an appropriate setting.

## Statistics

Descriptive statistics were used to assess the sample, which was analyzed using absolute numbers, percentages, averages and standard deviations. In order to calculate differences between the averages of the two groups, the Student’s *t* test for independent samples was used. The relationship among the SDQ total and factor scores of the quality of life (WHOQOL-BREF and AUQUEI), clinical impression of the inpatients, and psychopathology of the caregivers was assessed using the Pearson correlation coefficient (r). We considered the following magnitudes of correlation: very low (.00 to .19), weak (.20 to .39), moderate (.40 to .59), strong (from .60 to .79), and very strong (from .80 to 1.00) [42]. To calculate T scores for the quality of live (QOL) questionnaires, we first calculated the Z scores and used the normative scores by sex for the age group according to the normative values. The T scores were obtained by the following formula: T = 50 + 10Z, where the value 50 represents the normative average and 10 represents the standard deviation (SD). The QOL impairment was determined as being any value less than a standard deviation below the mean normative scores of the respective QOL scales (for both WHOQOL-BREF and AUQUEI). The significance threshold was considered at *p *< .05. All analyses were conducted using the SPSS program version 23.

## Results

The final sample consisted of 34 children from 25 patients. The age ranged from 4 for 17 years old (average was 10.8 ± 4.19). The majority (58.8%) of children was less than 12 years old and of female gender (52.9%). Most children and adolescents were children of hospitalized mothers and lived with their mothers (82.4%), siblings (58.8%), and fathers (47.1%) before hospitalization. Some of these children (17%) had been previously subjected to some previous mental health treatment. Their parents were mainly diagnosed with mood disorders (unipolar depression and bipolar disorder), and most of them were cared for by their mothers before the hospitalization. During the hospitalization, care was provided mainly by other relatives (41.2%) or by fathers (29.4%). The clinical and sociodemographic data are summarized in the Table 1.

**Table 1:** Sociodemographic and clinical data of the sample (n = 34) and their hospitalized parents

Variable	M ± SD or percentage
Female sex (%)	52.9
Age (M ± SD)	10.8 ± 4.19 [range from 4 to 17]
Age (%)	
< 12 years	58.8
≥ 12 years	41.1
Number of parents hospitalized (n)	25
Which parent hospitalized (%)	
Mother	85.3
Father	14.7
Age of the hospitalized parent (M ± SD)	38.9 ± 6.8
Age of the mother	38.2 ± 6.8
Age of the father	41.5 ± 9.1
Family income^a^—U$ (M ± SD)	1953 (2341)
Family income^a^ (%)	
Up to U$ 1.000,00	48
From U$ 1.000,00 to U$ 2.000,00	28
More than U$ 2.000,00	24
Lives with whom (%)	
Mother	82.4
Father	47.1
Siblings	58.8
Grandparents	14.7
Others	23.5
Number of people in the house (M ± SD)	3.93 ± 1.14
Years of study (Child)	9.41 ± 3.77
Previous treatment (Child %)	17.6
Parenteral psychiatric diagnosis^a^ (%)	
Unipolar depression	52
Bipolar disorder	24
Substance use/misuse	16
Personality disorder	4
Organic mental disorders	4
Caregiver before/during hospitalization (%)	
Mother	79.4/8.8
Father	11.8/29.4
Sibling	5.9/17.6
Another relative	11.8/41.2
Non-family caregiver	8.8/23.5

Family income calculated by basic salaries in Reais (R$ 937 or U$ 383; U$ 1.00 ≅  R$ 3.30)

^a^ Variables with missing values

Table 2 shows the average scores of SDQ, WHOQOL, AUQEI, the percentage of children with high risk of psychopathology, as well as the clinical features of caregivers and inpatient parents. According to the data from the SDQ, 38.3% of the children were at high risk for developing a psychiatric illness, including attention deficit hyperactivity disorder (38.2%), behavioral disorders (20.6%), and emotional disorders (17.6%). Of the offspring assessed in the present study, 41.2% were determined to be in situations of suffering or vulnerability and were recommended for psychiatric monitoring. These children were referred for outpatient psychiatric care when necessary. One child presented suicide ideation and was referred to an emergency department. Moreover, 61.8% of them presented impairment in their quality of life. In 70.6% of cases, the primary caregiver before admission was the inpatient, and they presented an average CGI of 5.2 (markedly ill). The average score of psychopathology of caregivers during parental hospitalization (as measure by PHQ) was 5.2 (mild to moderate distress) with high scores for anxiety.

**Table 2:** Clinical findings of inpatients offspring (n = 34), inpatient parents (n = 25) and caregivers (n = 25)

Variables children (n = 34)	M ± SD or percentage
SDQ—informant (M ± SD)	
Overall stress	14.0 ± 7.3
Emotional distress	4.1 ± 2.7
Behavioural difficulties	2.7 ± 2.7
Hyperactivity and concentration difficulties	4.4 ± 3.1
Difficulties getting along with other young people	2.6 ± 1.8
Kind and helpful behaviour	8.3 ± 2.1
Impact of any difficulties on the young person’s life	1.0 ± 1.3
Internalizing symptoms (M ± SD)	6.8 ± 3.7
Externalizing symptoms (M ± SD)	7.2 ± 5.1
SDQ—diagnostic predictions (% high risk)	
Any disorder	38.3
Emotional disorder	17.6
Behavioral disorder	20.6
Hyperactivity or concentration disorder	38.2
WHOQOLª (M ± SD)	
Physical	15.4 ± 2.4
Psychological	13.4 ± 2.5
Social	14.1 ± 4.4
Environmental	14.1 ± 2.4
Overall	15.0 ± 2.9
AUQEI^b^ (M ± SD)	44.9 ± 6.0
QOL—impairment (%)	61.8
Children referred for psychiatric evaluation (%)	
Already in treatment	17.6
Referred for psychiatric evaluation	41.2
Variables caregivers (n = 25)	
PHQ depression caregivers (M ± SD)	2.3 ± 1.7
PHQ anxiety—caregivers (M ± SD)	2.9 ± 2.0
PHQ-4 total—caregivers (M ± SD)	5.2 ± 3.4
PHQ-4 categories—symptoms in caregivers (%)	
None	20.8
Mild	33.3
Moderate	29.2
Severe	16.7
Variables inpatient parents (n = 25)	
Clinical Global Impression-Severity (CGI-S)—parent impatient (M ± SD)	5.2 ± 1.0
Primary caregiver is the inpatient (%)	70.6

*SDQ* Strengths and Difficulties Questionnaire total, *WHOQOL* World Health Organization Quality of Life questionnaire; *AUQUEI* Quality of Life Evaluation Scale, *PHQ* Patient Health Questionnaire

^a^ Used only for adolescents (> 12 and < 18 years; n = 13)

^b^Used only by children (from 4 to 12 years; n = 20)

The correlations between SDQ total and factors scores, internalizing and externalizing problems, and clinical variables of child and adolescents and their parents are presented in Table 3. SDQ total scores and some SDQ dimensions reached strong negative correlations between mental hospitalization of parents with domains of quality of life in adolescents, mainly in physical and social domains. In children, prosocial scores achieved a moderate positive correlation with quality of life. Scores of psychopathology in caregivers, particularly for anxiety, reached a weak to moderate positive correlation with several domains of SDQ, mainly with emotional problems, conduct problems, and internalizing symptoms.

**Table 3:** Correlations among Strengths and Difficulties Questionnaire (SDQ) total and factors scores, internalizing and externalizing problems, and clinical variables of child and adolescents and their parents

	SDQt	EMO	CON	HYPer	PEER	PROs	IMP	EXT	INT
QOLphyª	− .619*	− .510	− .500	− .293	− .458	.493	− .743**	− .419	− .595*
QOLpsyª	− .513	− .466	− .592*	− .233	.014	.670*	− .420	− .405	− .418
QOLsocª	− .630*	− .619*	− .635*	− .207	− .273	.371	− .709**	− .400	− .640*
QOLenvª	− .247	− .429	− .316	.214	− .459	.074	− .455	.053	− .523
AUQUEI^b^	.075	− .085	.052	.158	.109	.460*	− .196	.115	− .006
PHQdep	.244	.201	.233	.022	.281	− .045	.136	.138	.292
PHQans	.399*	.336	.395*	.115	.297	− .212	.176	.283	.401*
PHQtotal	.394*	.352*	.372*	.088	.329	− .124	.221	.243	.394*
CGIpar	.213	.132	.125	.102	.284	− .204	.223	.130	.242

*SDQt* Strengths and Difficulties Questionnaire (SDQ) total score, *EMO* SDQ Emotional problems scale, *CON* SDQ Conduct problems Scale, *HYPer* SDQ Hyperactivity scale, *PEER* SDQ Peer problems scale, *PROs* SDQ Prosocial scale, *IMP* SDQ impact scale, *EXT* externalizing problems, *INT* Internalizing problems, *QOLfhy* WHOQOL physical domain, *QOLpsy* WHOQOL psychological domain, *QOLsoc* WHOQOL social domain, *QOLanv* WHOQOL environmental domain, *AUQUEI* Quality of Life Evaluation Scale, *PHQdep* Patient Health Questionnaire-depression, *PHQans* Patient Health Questionnaire-anxiety, *PHQtotal* Patient Health Questionnaire total score, *CGIpar* Clinical Global Impression inpatient parent

^a^ Used only for adolescents (> 12 and < 18 years; n = 13)

^b^ Used only by children (from 4 to 12 years; n = 20)

* p < .05

## Discussion

Parental mental disorders have a dramatic impact on the next generation. In particular, offspring of parents with major mental disorders have an elevated risk of developing a mental disorder. Based on that assumption, the aim of this study was to evaluate the impact of parental mental illness on children of psychiatric inpatients. The children were evaluated through the perception of the caregiver during the hospitalization and their own perception and these evaluations were then correlated with clinical data of the hospitalized parent. We found that the offspring of inpatients presented high risk for psychopathology as well as impairment in the quality of life. A large proportion of the children was referred for specialized evaluation, especially those whose inpatient parent and/or caregiver during admission presented severe symptoms of psychopathology. As far as we could verify, this was the first study in Brazil evaluating the offspring of psychiatric inpatients.

Studies on children and adolescent psychopathology are relatively rare in low- and middle-income countries [3]. A large part of the research addressing the influence of parental psychopathology in offspring study adults [43–45]. Most of the studies to date have examined community samples. In a worldwide meta-analytic study, Polanczyk et al. determined that there was a 8.3% (in Africa) and 14.2% (in South America and Caribbean) prevalence of mental disorders in children and adolescents in the community [4]. In non-clinical samples of Brazilian children and adolescents, the prevalence of mental disorders range from 13% (in younger children) [9] to 30% (for common mental disorders in adolescents) [7]. In a high-risk cohort, Salum et al. reported mental disorder prevalence to be 19.9% of mental disorders from a random sample and 29.7% in the high-risk strata [46]. As such, the prevalence of 38.3% of mental disorders in our sample is higher than community non-clinical and high-risk samples. This result was higher than the 32% of psychopathology found in children of German parents with severe mental disorders [47]. This rate is also higher than the 23.7% prevalence of any psychopathology in children of patients with depression in the UK [25].

In our study, the hospitalized parent of 70.6% of the 34 children was their primary caregiver prior to psychiatric hospitalization. This may indicate that they were in the custody of parents that were potentially compromised in their care skills. Data from the UK show that at least a quarter of adults admitted to hospital settings (acute settings) have dependent children and between 50 and 66% of people with severe mental illness live with children under 18 years of age [48]. The intense relationship between children and seriously ill caregivers with psychiatric disorders often produces disorganized families and may lead to the development of pathologies in these children. The literature is extensive on the subject of growing with a mentally ill parent and the increased risk of persistent emotional and behavioral disorders in these children [25, 49–51]. Emotional and behavioral problems are related to low social competence [52]. In addition, the relationship with the child may be compromised, as studies report that parents with mental illness have problems with parenting in daily life, including difficulties in talking to children about their mental illness, maintaining discipline, and giving limits. Parental behavior can change due to disease symptoms or side effects of medications. Moreover, feelings of guilt, shame, and fear regarding adverse effects can also affect the parent’s relationship with the children [53]. Furthermore, when the primary caregiver is hospitalized, there may be an abrupt change in the dynamics of care of these children and the substitute caregiver does not always has a close link with them.

In addition to the mentally ill parent, we found that almost half of the children caregivers during the parent’s hospitalization had moderate (29.2%) to severe (16.7%) distress symptoms. Furthermore, the distress symptoms of caregivers were significantly associated with scores of emotional and conduct problems and internalizing symptoms. Thus, even when separated from their more psychiatric-diseased parent, half of these children were still exposed to caregivers (the other parent or other family member) with significant psychiatric symptoms. Studies have shown that when both parents are affected by psychopathology, the offspring have at least a double risk of psychopathology, behavior problems, or suicide [11, 17].

The quality of life (QOL) was impaired in 61.8% of our sample of children from psychiatric inpatients. Additionally, we found a significant negative association of high magnitude among several WHOQOL domains and emotional, conduct and internalizing problems in adolescents. Furthermore, was found a significant positive association of moderate magnitude between the Prosocial Scale and QOF in children. These results corroborate previous findings that parents with more serious illnesses are expected to have children with impaired quality of life, emotional distress, and behavior problems [47]. Although there are many questions about the term quality of life, and this term is considered by many authors to be difficult to evaluate [38], studies have shown that mentally ill children have a lower health-related quality of life (HRQL) than healthy or somatically ill children [47]. The effect of having a mentally ill parent on QOL may be related to mental distress and may evolve into more serious problems in the future.

The well-being of children of inpatients with mental disorders is a aspect that is not systematically collected by institutions, since the focus of the intervention remains centered on the inpatient. When the relative is hospitalized, it is an opportunity for the health service to protect and potentially strengthen the bond between the children their parents and promote the detection of mental problems and well-being of the children [54]. The results of our study indicate that there is a major need to evaluate and refer to the treatment of the children of inpatients who are often neglected due to the serious health situation of their main caregiver. Of the children evaluated in the present study, 17.8% were already in treatment, which may be considered a low rate for a population at risk. In addition, we found that another 41% of the children had some mental health problem that needed specialized evaluation, so they were referred to specialized professionals. Early intervention and prevention offer the possibility to avoid mental health problems in adults and improve personal well-being and productivity [3].

It was determined that in relation to parental diagnoses, unipolar depression was prevalent in 52% of hospitalized relatives. This is often an incapacitating psychiatric illness that leads to difficulties in self-care and self-management. These difficulties can have repercussions on family relationships and impact the lives of the children. Descendants of parents with major depression disorders have higher rates of psychiatric disorder than children of parents who are not affected. Children with unipolar depression are more likely to have a parent with unipolar depression than other parental diseases [55]. Common parenting styles among parents with depression, such as low levels of child monitoring, may also play a role in the development of childhood mental health problems [13]. Hammen [56] found that the patterns of parenting established by depressed mothers can be learned by their children, who later parent the same way and maintain negative patterns of interaction over generations. Most studies examining parental mental illness have assessed adults with depressive symptoms and have found a 3–4 fold increase in symptomatology in children compared to controls [12]. The type of psychiatric illness, severity, associated impairments, as well as the degree of support from other family members seems to influence this risk. Compared with children of healthy parents, those living with serious mental illness may also be exposed to greater material deprivation, increased adult responsibilities and self-care, and increased risk of maltreatment and neglect [47].

The adequate identification of children at risk allows a quick referral for care. The possibility of intervention and follow-up of these children could reduce the suffering and psychiatric symptoms in children and adolescents, as shown by international strategies and studies like Preventive Basic Care Management (PBCM) [55], and Let’s Talk in Australia [57], which are programs that aim to identify if the children of patients with mental disorders situations need intervention and to promote well-being and quality of life. Screening and early intervention in children from high-risk psychopathology groups is a challenge that needs to be addressed. In tertiary environments, the first step is to identify patients with children, which is often difficult because they are not questioned and such information is not recorded in medical records. This is a subject that is rarely touched upon in medical practice and is still stigmatized because it is very difficult for parents to talk about these problems with their doctors [29]. There is evidence that both children and parents benefit from adequate identification, as this may influence the treatment and recovery of psychiatric illness. Thus, identifying and supporting an individual’s parenting role can provide hope, a sense of action, self-determination, and meaning, all aligned with a recovery approach. For those parents with a mental illness, parental support can provide a sense of competence, belonging, identity, hope and meaning that is well aligned with the concept of personal recovery [57]. In addition to the arguments of how societal costs can be reduced by early intervention, there is also ethical responsibility to the most vulnerable young people, who can have their full developmental potential thwarted [3]. We still have a lot to do for these children and adolescents in order to identify risk situations, try to alleviate suffering and prevent new diseases.

This study has several limitations. First, our sample size is very small, which excluded the ability to use several analytical strategies. Our sample size suffered a lot of losses due to logistical difficulties (i.e., location of caregivers, difficulties of accessing them to the hospital, and refusal of many parents to allow the evaluation of their children) and the non-routinization of this type of assessment in the unit. However, we believe that the data presented is significant and may still be underestimate the effect of having a parent with mental illness on the well-being of a child. Nevertheless, we are implementing an evaluation routine for children of inpatients based this study. Second, the sample consisted of patients and their children from only one psychiatric unit, which decreases its external validity. However, since screening programs are not usually used in our environment, we believe that our data is indicative of a much larger problem, and replications will be required. In addition, short hospitalizations, with less than a week, also made some evaluations unviable. Finally, the data on psychopathology in children were collected from their caregivers, which may have influenced the evaluation, since many of them also exhibited psychiatric symptoms. However, quality of life assessments were conducted directly with children and adolescents, allowing a more direct measure of the impact of parental symptoms in their lives.

This work reinforces the importance of the routine screening of psychopathology in children of hospitalized psychiatric patients. Several barriers related to economic factors, integration of the health system, inadequate insurance coverage and unavailability, and overloading of the teams make it difficult for children and adolescents to access health services [58]. The development of assistance is also hampered by lack of government policy, inadequate funding, and a dearth of trained professionals [3]. Thus, we believe that the insertion of the evaluation routine of children of patients can be an important step for the identification of vulnerable children and adolescents stresses the need for institutions and governments to construct public policies that prioritize this issue.
